# Supportive interventions to enhance the mental health of children - an under-researched field

**DOI:** 10.1186/1753-2000-3-31

**Published:** 2009-10-14

**Authors:** Lutz Goldbeck

**Affiliations:** 1 Department of Child and Adolescent Psychiatry/Psychotherapy, University Hospital Ulm, Steinhövelst 5, 89075 Ulm, Germany

## Editorial

Child mental health is a goal of high priority for parents in post-modern society, as many stressors may threaten the psychological well-being of the few children that are born in the so-called developed countries. By noting the high prevalence of mental disorders during childhood and adolescence parents are aware how fragile the psychosocial development of their child may be. Aiming at these parental concerns, many programs claiming to improve the mental health of children are marketed by different vendors outside of the professional mental health services. Such programs comprise sports activities, mental training programs, dietary programs, and other complementary interventions.

According to their professional guidelines, psychiatrists and psychotherapists are used to base their interventions on evidence, e.g. randomized controlled studies showing the efficacy and effectiveness of a specific intervention and the absence of harm caused by this intervention. Outside of the mental health field, many programs and interventions are offered by claiming to maintain or improve the mental health of children; however, no empirical evidence is presented. Parents seek the advice of therapists about the usefulness of such programs to support their child, and even more parents may expect that such interventions may be equivalent to professional child psychotherapy or child psychiatry, if their child has behavioural or emotional symptoms. On the other hand, therapists may tend to counsel patients to utilize additional programs and activities, thus suggesting a positive effect on mental health, social competence, etc. Usually the impact of these interventions on behavior and psychosocial development is not investigated.

Martial arts are an example of an activity claimed to improve child mental health, but whose possible therapeutic benefits remain obscure. The study of Strayhorn and Strayhorn [1] published today in *Child and Adolescent Psychiatry and Mental Health *offers an innovative empirical approach to measuring the impact of participation in martial arts on child mental health. One would expect benefits of such an intervention for a variety of children, regardless of specific diagnoses, therefore the authors' decision to conduct a secondary analysis of a large population-based longitudinal cohort study guarantees results of high external validity. Based on the Early Childhood Longitudinal study, the authors were able to analyse teacher reports of several thousand children's classroom behavior at two time points - 3^rd ^grades and 5^th ^grades - in relation to previous participation in martial arts. The proportion of about 6 to 7 per cent of U.S. children participating in martial arts training is striking. Although martial arts as defined in this study is a heterogeneous type of intervention and the dosage of this activity is not controlled for, the external validity of the study is high due to the large and quite representative sample. The study design is innovative for a program evaluation, as it used epidemiological data for a program evaluation. The results indicate that effects of martial arts as provided in the U.S. on the children's classroom-behavior are absent, at least from the teachers' perspective. It can be concluded from this study, that the proposed broadband impact of martial arts training cannot be demonstrated.

This study provides preliminary results on the effectiveness of martial arts for the general mental health of children. More studies are necessary to determine the effectiveness for children with special needs. E.g., differential effects might be found for healthy children, children with internalizing disorders, and children with externalizing disorders. These questions can be optimally addressed in prospective, randomized controlled studies of treatment as usual alone vs. combined treatment as usual and participation in martial arts vs. no treatment.

Further studies of complementary or supportive programs and interventions are needed to inform clinicians and families on the usefulness of specific activities or programs to enhance the psychosocial development of children. Research of this kind builds bridges to the daily challenges that are faced in routine clinical work with children, adolescents and their families. Therefore, the editors of CAPMH would like to encourage researchers from all disciplines to submit papers relevant to this still under-investigated area.
